# Excessive Daytime Sleepiness Is Associated with Changes in Salivary Inflammatory Genes Transcripts

**DOI:** 10.1155/2015/539627

**Published:** 2015-03-22

**Authors:** Matthew S. Thimgan, Cristina Toedebusch, Jennifer McLeland, Stephen P. Duntley, Paul J. Shaw

**Affiliations:** ^1^Department of Anatomy and Physiology, Washington University School of Medicine, St. Louis, MO 63110, USA; ^2^Department of Biological Sciences, Missouri University of Science and Technology, 400 W. 11th Street, Rolla, MO 65401, USA; ^3^Department of Neurology, Washington University School of Medicine, St. Louis, MO 63110, USA; ^4^Critical Care, Pulmonary and Sleep Associates, 274 Union Boulevard, Suite 110, Lakewood, CO 80228, USA

## Abstract

Excessive daytime sleepiness (EDS) is a ubiquitous problem that affects public health and safety. A test that can reliably identify individuals that suffer from EDS is needed. In contrast to other methods, salivary biomarkers are an objective, inexpensive, and noninvasive method to identify individuals with inadequate sleep. Although we have previously shown that inflammatory genes are elevated in saliva samples taken from sleep deprived individuals, it is unclear if inflammatory genes will be elevated in clinical populations with EDS. In this study, salivary samples from individuals with sleep apnea were evaluated using the Taqman low density inflammation array. Transcript levels for 3 genes, including *prostaglandin-endoperoxide synthase 2 (PTGS2)*, were elevated in patients with sleep apnea. Interestingly, *PTGS2* was also elevated in patients with EDS but who did not have sleep apnea. These data demonstrate the feasibility of using salivary transcript levels to identify individuals that self-report excessive daytime sleepiness.

## 1. Introduction

Inadequate sleep is a pervasive problem in today's society. Insufficient sleep leads to decreased cognitive performance [1], increased sleepiness [2–4], reduced productivity [5], and increased traffic accidents [6]. Moreover, inadequate sleep increases the susceptibility of individuals to adverse health outcomes, including cardiovascular deficits [7], increased immune challenges [8, 9], longer recovery times after injury [10], increases in sympathetic tone [11, 12], and reduced lifespan [7]. Given the number and severity of consequences that accompany inadequate sleep, it would be helpful to have a simple and reliable test to identify vulnerable individuals before they experience adverse consequences.

One approach to accomplish this objective has been to test candidate biomarkers to determine whether they are consistently altered in subjects with inadequate or insufficient sleep. Biomarkers are objective, often endogenous factors that report changes in body chemistry and correlate with either disease state or the severity of the disease. Several biomarkers, including eyelid closures [13] and balance [14], as well as biochemical markers from blood [15, 16], cerebral spinal fluid [17–19], and breath analytes [20] have been evaluated as candidate biomarkers of disrupted sleep. Although each of these approaches has had limited success, assessment tools that can be used in real-world settings are not yet widely available. With that in mind, we have hypothesized that saliva, as a rich source of analytes, can be mined to identify biomarkers of sleepiness or inadequate sleep. Saliva is particularly well suited for monitoring sleepiness since it is a readily accessible biological fluid that can be easily collected using noninvasive procedures. Indeed, we have shown that transcripts for *α-amylase*,* Filamin-A*,* maleic enzyme*,* integrin*, *αM*, and* integrin*, *α5,* are all elevated in saliva samples following sleep deprivation [21–24]. Increased levels of salivary *α*-amylase activity have also been shown to correlate with increased sleepiness and decreased cognitive performance in an independent study [25]. However, since endogenous factors are frequently modulated by a variety of physiological conditions (stress, circadian time, etc.), test of sleepiness should be comprised of a panel of independent analytes. Several studies have found that serum markers of inflammation are elevated in populations of individuals with sleep disorders [9]. For example, serum levels of interleukin 6 (IL-6) [26], interleukin 8 (IL-8) [27, 28], tumor necrosis factor-*α* (TNF-*α*) [29], C-reactive protein (CRP) [30], intracellular adhesion molecule (ICAM) [31], selectins [32], and vascular cell adhesion molecule (VCAM) [31] have all been shown to increase in multiple populations of patients with sleep apnea. Therefore, we wanted to determine if levels of salivary inflammation transcripts could be used to identify sleepiness in a clinical population, individuals diagnosed with sleep apnea.

## 2. Materials and Methods

### 2.1. Subjects and Samples

8 controls, 14 patients confirmed to have sleep apnea, and 18 patients that were suspected to have sleep apnea during their initial screen but did not exhibit sleep apnea during their overnight assessment (sleepy) were evaluated for salivary transcript levels. Both the sleep apnea group and the “sleepy” group were referred to the Washington University Sleep Laboratory due to excessive sleepiness. Samples were taken from patients at ~9 pm after they had arrived at the sleep lab and prior to beginning polysomnography. The apnea/hypopnea index (AHI) was determined using polysomnography (PSG) as previously described [33]. Subjects were free from psychiatric disorders and prescription medicines [24]. Each subject was administered the Epworth Sleepiness Scale (ESS) and their body mass index (BMI) was calculated. BMI was unavailable for one subject in the “sleepy” group. Consent was received from all participants and all protocols were approved by the Washington University School of Medicine Institutional Review Board.

### 2.2. Low Density Arrays

Saliva was taken and cDNA was generated according to the protocol previously described [24]. Briefly, saliva was obtained when subjects chewed on a salivette (Sarstedt, Newton, NC). One mL of RNAlater (Life Technologies, Grand Island, NY) was added to the salivette and immediately frozen on dry ice and stored at −80°C. At the time of transcript analysis, salivettes were thawed and the saliva-RNAlater extracts were extracted by centrifugation. RNA was purified from cell-free lysates and reverse-transcribed using Superscript III (Life Technologies, Grand Island, NY) according to the manufacturer's instructions. Equal amounts of cDNA were loaded into an inflammation Taqman low density array (LDA) (Life Technologies, Grand Island, NY) and Taqman based qPCR was carried out using the 7600 real-time PCR system (Applied Biosystems, Foster City, CA). The LDAs were normalized to 18S RNA. Fold changes were determined using the method described in [34], known as the comparative C_T_ method. Transcript levels for the gene of interest were first normalized within subject to 18S RNA.

### 2.3. Statistics

All demographic data is presented as average ± standard deviation. For transcript analysis, if a transcript was not detected in more than 77% of the group, the transcript was deemed undetectable and not analyzed. For all detectable genes, transcript levels for each subject were determined as a fold change from the average levels of the control group. Fold changes were converted to a log_2_ scale. From the log data, average and standard error were calculated. Groups of interest were compared to control levels using a two-tailed, unpaired Student's* t*-test and the significance level was set at 0.05. *P* values were corrected for multiple comparisons using the false discovery method (FDR) with a FDR level of 5% [35]. Transcript levels are presented as a geometric mean and standard error [36]. For ease of presentation, group data, including average, upper, and lower bounds, were then converted back to standard units by raising 2 to the power of the calculated value to obtain fold change values.

## 3. Results

### 3.1. Demographic Data for Patients with Sleep Apnea

We evaluated 14 patients with sleep apnea (11 male and 3 female) and 8 control subjects (5 male and 3 female) for salivary transcriptional changes associated with sleep apnea. Control subjects did not have any prior existing sleep or mental disorders, were not on prescription medications, had not consumed caffeine, and had not eaten 1 hour prior to providing a saliva sample. Subject age was not different between the two groups (Table 1). The subjects with sleep apnea had an average AHI of 48.5 ± 23.6 (mean ± S.D.). The subjects with sleep apnea had a significantly higher body mass index (BMI) than the controls. Consistent with previous results, subjects with sleep apnea also reported a higher score on the Epworth Sleepiness Scale.

### 3.2. Evaluation of Salivary Biomarkers Associated with Inflammation in Patients with Sleep Apnea

We evaluated salivary transcript levels from subjects with sleep apnea and control subjects using low density arrays. The LDA platform simultaneously evaluates the levels of 96 RNA species. Of the 96 genes tested, 21 transcripts were detected in 11 or more sleep apnea patients. Fold changes from control and *P* value for each gene are presented in Table 2. The *P* value listed was determined using a two-tailed Student's* t*-test comparing the controls to sleep apnea for all analyzed genes present in saliva. We performed a correction for multiple comparisons using a FDR set at 5%, which yielded a new *P* value equivalent to *α* = 0.0071 [35]. Compared to controls, 3 transcripts exhibited significant changes in patients with sleep apnea (Table 2),* ANXA1*, *β2M*, and* PTGS2* (see Table 2 for abbreviations). These results indicate that several inflammatory transcripts are increased in patients with sleep apnea.

### 3.3. Demographic Data for Patients with an Elevated ESS but without Sleep Apnea

To assess whether the increased inflammatory markers were due primarily to hypoxia or whether they might be more closely associated with increased sleepiness, we evaluated transcriptional changes in an independent group of people that entered the sleep lab suspected of having sleep apnea due to initial screening but ultimately had a normal AHI as determined by PSG (sleepy). The “sleepy” group was composed of 6 males and 12 females. By definition, all individuals in this group had an AHI less than 5. However, all subjects reported ESS scores significantly higher than controls and similar to the subjects with sleep apnea (Tables 1 and 3). “Sleepy” subjects had a similar age to controls but an increased BMI.

### 3.4. Salivary Transcript Levels in “Sleepy” People

Twenty-one transcripts were detected in the saliva of 14 or more “sleepy” patients. As above, to facilitate comparisons with the apnea patients reported in Table 2, Table 4 includes transcripts for fold changes for each of the 21 transcripts. After and FDR correction for multiple comparisons (*α* = 0.005), 2 transcripts were significantly changed in the “sleepy” population,* CASP1* and* PTGS2*.* PTGS2* can be considered an independent replicate of the apnea results and may deserve further consideration. Thus, inflammatory transcripts are elevated under conditions of EDS.

### 3.5. Transcriptional Changes Are Not due to Elevated BMI

**Figure 1:**
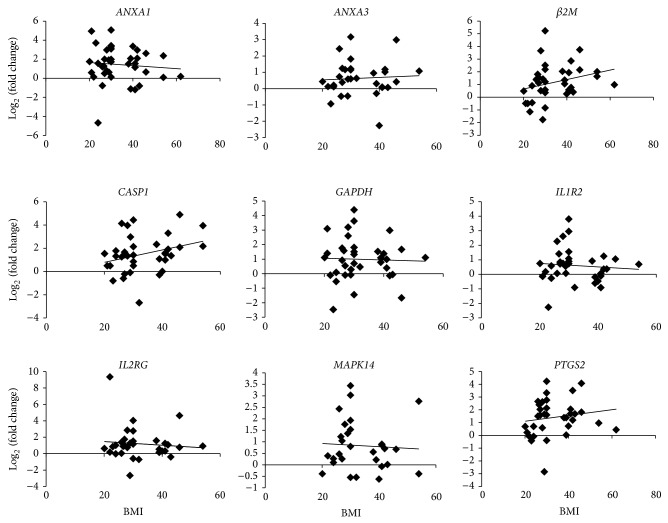
Correlation of transcript levels with BMI. The transcript levels were plotted against the log values of the transcript levels for each of the transcripts that were significant in the sleep apnea or “sleepy” groups. In addition the transcripts with the lowest *P* value were plotted to determine if there was a significant correlation with increased weight. For each transcript, there was no significant correlation with increased BMI.

One possible explanation for elevated inflammatory transcripts is that both the sleep apnea and “sleepy” patients have a significantly higher BMI compared to controls. If the observed relationship is due solely to BMI, then patients with high BMI should have increased levels of inflammatory transcripts compared to patients with lower BMI. Similarly, we should detect a significant positive correlation between BMI and the level of salivary transcripts. To test this hypothesis we discretized the data by placing the subjects with a low BMI (≤30; *n* = 24) into a single group and comparing them to subjects with a high BMI (>38; *n* = 16). As seen in Table 5, transcripts were not significantly different between subjects in the lower BMI group compared to their counterparts with a higher BMI. In addition, we evaluated the relationship between BMI and transcript levels using a Pearson correlation and found no significant correlations. Examples of transcript expression levels for the genes with the lowest *P* values are plotted in Figure 1 as a function of BMI. These data indicate that, in this dataset, BMI does not account for increases in transcript levels in these patients.

## 4. Discussion

In this paper, we report that salivary transcript levels of inflammatory genes are increased both in patients with sleep apnea and in an independent cohort of patients who self-identified as sleepy and were referred to the sleep lab suspected of having sleep apnea. We have previously used these same human low density arrays as a discovery tool to identify genes that are modified following acute sleep deprivation in healthy adults [24]. In that study, we reported that* integrin, *α*M* (*ITGAM*), and* annexin A3* (*ANXA3*) were significantly increased following 24 h and 30 h of waking. Interestingly, neither* ITGAM* nor* ANXA3* were altered in either sleep apnea or “sleepy” subjects suggesting that they may be better suited for detecting acute sleep loss than for identifying patients with chronic sleep disruption. In contrast, salivary* PTGS2* was significantly increased in both sleep apnea patients and in the “sleepy” cohort but was not affected by acute sleep deprivation [24]. Thus, these data support our previous proposal that a panel of biomarkers will be required to accurately identify sleep deprived individuals and further suggest that different sets of biomarkers may be required to distinguish between acute and chronic sleep disruptions.

Sleep apnea has been particularly difficult to diagnose and treat and the incidence of sleep apnea has been difficult to determine. Estimates of sleep apnea have ranged from as low as 2–4% to as high as 24% of middle aged men [37], although other studies have estimated the likelihood of sleep apnea to be much higher still [38, 39]. Moreover, it has been suggested that sleep apnea is likely to be substantially underdiagnosed [38, 39]. Indeed, years may transpire between the onset of sleep apnea and the eventual diagnosis [40]. As the population ages and becomes more obese, the prevalence of sleep apnea is expected to increase even further [7, 41, 42]. During these intervening years, the severity and consequences of the sleep apnea may also increase [41, 42]. The associated consequences of sleep apnea include increased daytime sleepiness [3] and an increased likelihood of automobile accidents [6]. Moreover, sleep apnea has been associated with a host of cardiovascular complications [43, 44] and increased rates of all-cause mortality [7]. Sleep apnea has also been associated with learning impairments [45]. The current gold standard of treatment, continuous positive airway pressure (CPAP), may be able to reverse some of the consequences of sleep apnea, such as sleepiness [2] and restoration of typical sleep stages [2], and may lower blood pressure [46] but the literature is less conclusive whether cognitive function is improved [4, 47]. Thus, a clinical goal is to have patients begin treatment for sleep apnea as soon as possible.

A simple cost-effective biomarker that can be easily used at the point of care may be a useful tool to convince people to go to the sleep lab for a more precise diagnosis and treatment. Since biomarkers are both objective and quantifiable, they are well suited for persuading an individual that they may have an underlying affliction that needs greater attention. In that regard, it is important to note that the well-known relationship between inflammation and sleep deprivation is an advantage since many physicians are likely to be familiar with this relationship and thus more willing to incorporate such a biomarker into their treatment practices. That is, numerous inflammatory markers, including IL-6 [20, 26, 48], IL-8 [27, 28, 48], TNF-*α* [26], CRP [49, 50], monocyte chemoattractant protein-1 (MCP-1) [27], ICAM [27, 51], selectins [51], VCAM [51], nitric oxide [52], and isoprostane [20], are elevated in body fluids of patients with sleep apnea. Moreover, proteomics have identified protein biomarkers in urine [53]. Interestingly, inflammatory markers have also been associated with sleep deprivation in serum [54, 55] and saliva in healthy adults [24]. In this study, transcript levels for* PTGS2* were elevated in people with inadequate sleep. Our results parallel findings in mice in which transcript levels for PTGS2 are elevated after acute sleep deprivation [56].* PTGS2*, along with* PTGS1*, is a critical enzyme in prostaglandin synthesis. Prostaglandins initiate a set of molecular cascades that result in an inflammatory response.* PTGS2* is induced by several stimuli including proinflammatory signals and converts arachidonic acid to prostaglandin H2 [57]. Thus,* PTGS2* plays a role in the inflammatory pathway in addition to being a candidate biomarker for EDS.

It is interesting to note that many studies compare sleep apnea patients to healthy controls. Although this approach has been quite successful, our data suggest that other patients with chronic sleep disruption may also experience some of the same outcomes as patients with sleep apnea. If this turns out to be the case, more generally, assessing chronically sleepy individuals along with patients that have sleep apnea may facilitate the identification of precise deficits that are due specifically to sleep loss rather than other features of sleep apnea, such as hypoxia [48]. Nonetheless, our results emphasize that* PTGS2* cannot distinguish between sleepy patients with and without sleep apnea. While this may seem disappointing, we believe that any diagnosis of a sleep disorder should be made by a qualified sleep physician after fully examining the patient. Indeed, it may not be possible to identify a biomarker that is specific to a single sleep disorder. One example of a promising biomarker is cerebrospinal fluid (CSF) levels of hypocretin-1/orexin (HCRT), which has emerged from research in the narcolepsy field [18, 58]. CSF levels of hypocretin-1/orexin are lower in narcoleptic patients. But for patients without cataplexy, decreased CSF levels of HCRT are not as predictive, even in combination with the genetic marker DBQ. Moreover, decreases in CSF levels of HCRT are also associated now, being associated with other diseases, including dementia with Lewy Body [59] and Alzheimer's disease [60] and Kleine-Levin syndrome [58, 61]. This example illustrates the difficulty of a single biomarker with a given sleep disorder. Nonetheless, a biomarker that can help primary physicians to identify sleepy individuals may be extremely useful for ensuring that they get the appropriate care in a timely manner.

Our protocol has two aspects that may be useful for the real-world application of biomarkers to identify patients with sleep disorders. First, acquiring saliva is a noninvasive procedure and does not, for example, require a private location for sample collection (e.g., urine) or specialized skills to acquire samples (e.g., blood). As a consequence, salivary biomarkers can be readily used both on the roadside and in a doctor's office with equal effectiveness. A second advantage of our protocol is our focus on salivary RNA coupled with the LDA platform for the rapid detection of 96 transcripts simultaneously. LDAs may be an effective way to screen multiple targeted transcripts at once without the burden that NextGen sequencing frequently demands. One advantage of using transcript levels as the biomarker is that nucleotide probes can be generated quickly and are very specific for the target intended as opposed to having to design an antibody against a protein target. Given human variability, the different roles that genes can play throughout the body (pleiotropy), and the likelihood that a given analyte will be induced by a variety of environmental situations, it is likely that no single biomarker will be ideal for reliable diagnosis of sleep apnea. Nonetheless, our data suggest that* PTGS2* may be a particularly good candidate for inclusion in a panel of biomarkers.

There are two potential weaknesses of our study. The first is the sample size of our groups. Low sample sizes may identify significant differences within the subgroup that does not generalize to the rest of the population. In this study, we have tried to balance the difficulty of completing discovery experiments with the expense of a human study. Although our sample size was low for sleep apnea subjects, we were able to confirm the changes in* PTGS2* in a separate cohort of “sleepy” patients. This confirmation adds to our confidence that* PTGS2* is a good candidate for followup studies. A second weakness of this study is that we were not able to control for the levels of BMI between our control subjects and patients with sleep apnea or the “sleepy” cohort. To address this issue, we discretized the subjects into groups with low or high BMI. Means testing between the two groups indicated the subjects with low and high BMI were not different from one another. Moreover, Pearson correlation between BMI and transcript levels did not reveal any significant associations. Finally, when examined as a whole, we could find no general trend of inflammatory genes being elevated in subjects with higher BMI. Thus, while BMI is a potential confounder, it did not appear to unduly influence the results of this study.

Biomarkers have the capacity to serve an important need in maintaining human health. They provide an objective insight into the goings-on of the body. In particular, there is a need to identify individuals with sleep problems that result in EDS. Given the difficulty in identifying individuals, the consequences of sleep apnea and other sleep disorders, and the potential that therapy possesses, objective biomarkers can be a way to convince people to go to the sleep lab for diagnosis. We have demonstrated the feasibility of using saliva to identify changes in inflammatory transcripts that correlate with the presence of EDS. Saliva is an ideal source of biofluid for a potential source of biomarkers. Obtaining saliva is a noninvasive and relatively inexpensive process. Further experiments in larger and independent populations will determine the precision and validity of these biomarkers, but these experiments demonstrate that saliva has the potential to be objective biomarkers to identify subjects with EDS.

## Figures and Tables

**Table 1:** Demographics for controls and sleep apnea.

	Controls (*n* = 8)	OSA (*n* = 14)
Gender	5M, 3F	11M, 3F
Age	40.8 ± 8.9	49.0 ± 12.2
AHI	—	48.5 ± 23.6
BMI^*^	25.7 ± 4.4	34.6 ± 7.1
ESS^*^	3.9 ± 2.7	10.8 ± 4.3

Ave. ± Std. Dev.; ^*^ *P* < 0.05.

**Table 2:** Salivary transcript levels in patients with sleep apnea compared to controls.

Gene	Abbreviation	Sleep apnea fold Δ from control^a^	*P* value
*β*-actin	ACTB	1.43 ± 0.38	0.191
Arachidonate 5-lipoxygenase	ALOX5	1.39 ± 0.36	0.238
Arachidonate 12-lipoxygenase	ALOX12	1.56 ± 0.69	0.404
Annexin A1	ANXA1	4.62 ± 1.15	0.003^b^
Annexin A3	ANXA3	1.26 ± 0.38	0.444
Annexin A5	ANXA5	2.05 ± 0.92	0.168
*β*-2-microglobulin	B2M	2.65 ± 0.70	0.007^b^
Caspase 1, apoptosis-related cysteine peptidase	CASP1	2.57 ± 0.81	0.025
Glyceraldehyde-3-phosphate dehydrogenase	GAPDH	2.66 ± 0.81	0.019
Intercellular adhesion molecule 1	ICAM1	0.44 ± 0.13	0.123
Interleukin 1 receptor, type II	IL1R2	1.62 ± 0.41	0.143
Interleukin 2 receptor, gamma	IL2RG	2.36 ± 0.72	0.054
Integrin, alpha M	ITGAM	1.50 ± 0.61	0.376
Integrin, beta 2	ITGB2	1.47 ± 0.31	0.167
Mitogen-activated protein kinase 1	MAPK1	1.72 ± 0.66	0.401
Mitogen-activated protein kinase 3	MAPK3	1.30 ± 0.41	0.585
Mitogen-activated protein kinase 14	MAPK14	1.85 ± 0.53	0.089
Phosphodiesterase 4B, cAMP-specific	PDE4B	1.49 ± 0.21	0.168
Prostaglandin-endoperoxide synthase 2 (prostaglandin G/H synthase and cyclooxygenase)	PTGS2	3.73 ± 0.98	0.002^b^
Tumor necrosis factor receptor superfamily, member 1A	TNFRSF1A	1.32 ± 0.20	0.206
Tumor necrosis factor receptor superfamily, member 1B	TNFRSF1B	1.07 ± 0.44	0.900

^a^Mean ± SEM.

^b^Significant difference using 5% FDR post hoc test.

**Table 3:** Demographics for “sleepy” subjects and controls.

	Controls (*n* = 8)	Sleepy (*n* = 18)
Gender	5M, 3F	6M, 12F
Age	40.8 ± 8.9	44.1 ± 14.4
AHI	—	4.7 ± 5.2
BMI^*^	25.7 ± 4.4	34.2 ± 10.8
ESS^*^	3.9 ± 2.7	11.0 ± 5.0

Ave ± Std. Dev.; ^*^ *P* < 0.05.

**Table 4:** Salivary transcript levels in “sleepy” patients compared to controls.

Gene	Abbreviation	Sleepy people fold Δ from control^a^	*P* value
*β*-actin	ACTB	1.86 ± 0.47	0.033
Arachidonate 5-lipoxygenase	ALOX5	1.30 ± 0.46	0.472
Arachidonate 12-lipoxygenase	ALOX12	0.53 ± 0.33	0.408
Annexin A1	ANXA1	2.86 ± 1.37	0.099
Annexin A3	ANXA3	2.64 ± 0.85	0.010
Annexin A5	ANXA5	2.29 ± 0.76	0.054
*β*-2-microglobulin	B2M	3.33 ± 1.34	0.013
Caspase 1, apoptosis-related cysteine peptidase	CASP1	5.36 ± 2.07	0.001^b^
Glyceraldehyde-3-phosphate dehydrogenase	GAPDH	2.35 ± 0.88	0.068
Intercellular adhesion molecule 1	ICAM1	0.61 ± 0.33	0.441
Interleukin 1 receptor, type II	IL1R2	2.04 ± 0.75	0.092
Interleukin 2 receptor, gamma	IL2RG	3.53 ± 2.25	0.118
Integrin, alpha M	ITGAM	1.64 ± 0.65	0.292
Integrin, beta 2	ITGB2	1.84 ± 0.52	0.102
Mitogen-activated protein kinase 1	MAPK1	1.55 ± 0.65	0.510
Mitogen-activated protein kinase 3	MAPK3	1.06 ± 0.42	0.921
Mitogen-activated protein kinase 14	MAPK14	2.33 ± 0.77	0.057
Phosphodiesterase 4B, cAMP-specific	PDE4B	1.69 ± 0.69	0.259
Prostaglandin-endoperoxide synthase 2 (prostaglandin G/H synthase and cyclooxygenase)	PTGS2	3.80 ± 1.34	0.005^b^
Tumor necrosis factor receptor superfamily, member 1A	TNFRSF1A	1.87 ± 0.62	0.096
Tumor necrosis factor receptor superfamily, member 1B	TNFRSF1B	1.80 ± 0.88	0.322

^a^Mean ± SEM.

^b^Significant difference using 5% FDR post hoc test.

**Table 5:** Comparison of transcriptional changes in high and low BMI subjects.

Gene	Abbreviation	BMI < 30	BMI > 30	*t*-test
		fold Δ^a,b^	fold Δ^a,c^	
*β*-actin	ACTB	1.39 ± 0.29	1.65 ± 0.40	0.438
Arachidonate 5-lipoxygenase	ALOX5	1.46 ± 0.33	0.97 ± 0.28	0.117
Arachidonate 12-lipoxygenase	ALOX12	1.25 ± 0.47	0.48 ± 0.32	0.058
Annexin A1	ANXA1	3.06 ± 1.25	2.33 ± 1.03	0.529
Annexin A3	ANXA3	1.68 ± 0.40	1.63 ± 0.61	0.913
Annexin A5	ANXA5	1.74 ± 0.58	2.17 ± 0.75	0.519
*β*-2-microglobulin	B2M	1.95 ± 0.66	3.12 ± 0.91	0.156
Caspase 1, apoptosis-related cysteine peptidase	CASP1	2.68 ± 0.90	3.05 ± 1.42	0.741
Glyceraldehyde-3-phosphate dehydrogenase	GAPDH	2.14 ± 0.72	1.94 ± 0.57	0.771
Intercellular adhesion molecule 1	ICAM1	0.61 ± 0.22	0.62 ± 0.36	0.982
Interleukin 1 receptor, type II	IL1R2	1.88 ± 0.53	1.24 ± 0.34	0.153
Interleukin 2 receptor, gamma	IL2RG	2.65 ± 1.37	2.20 ± 0.93	0.713
Integrin, alpha M	ITGAM	1.56 ± 0.48	1.25 ± 0.51	0.537
Integrin, beta 2	ITGB2	1.52 ± 0.35	1.54 ± 0.38	0.949
Mitogen-activated protein kinase 1	MAPK1	1.77 ± 0.83	1.20 ± 0.37	0.355
Mitogen-activated protein kinase 3	MAPK3	1.41 ± 0.36	0.90 ± 0.34	0.156
Mitogen-activated protein kinase 14	MAPK14	2.18 ± 0.60	1.51 ± 0.45	0.211
Phosphodiesterase 4B, cAMP-specific	PDE4B	1.44 ± 0.36	1.44 ± 0.55	0.999
Prostaglandin-endoperoxide synthase 2 (prostaglandin G/H synthase and cyclooxygenase)	PTGS2	2.43 ± 0.84	3.42 ± 1.13	0.338
Tumor necrosis factor receptor superfamily, member 1A	TNFRSF1A	1.47 ± 0.36	1.40 ± 0.33	0.841
Tumor necrosis factor receptor superfamily, member 1B	TNFRSF1B	1.72 ± 0.66	0.86 ± 0.36	0.099

^a^Mean ± SEM.

^b^ *n* = 15–24.

^c^ *n* = 11–15.
